# Carbon dots in tissue repair: from cell behavior to microenvironment remodeling

**DOI:** 10.3389/fbioe.2026.1912437

**Published:** 2026-10-05

**Authors:** Xumin Li, Junsi Luo, Shuangjiang Li

**Affiliations:** 1 School of Stomatology, Stomatological Hospital, Southern Medical University, Guangzhou, China; 2 Hunan Key Laboratory of Oral Health Research, Hunan Clinical Research Center of Oral Major Diseases and Oral Health, Xiangya Stomatological Hospital, Xiangya School of Stomatology Central South University, Changsha, China; 3 Department of Stomatology, Changsha Stomatological Hospital, Changsha, China

**Keywords:** antioxidation, carbon dots, immunoregulation, repair cells, tissue repair

## Abstract

Tissue repair is essential for restoring structural integrity and function after injury. Carbon dots (CDs) have garnered considerable interest in biomedicine due to their high specific surface area, excellent water solubility, biocompatibility, and facile doping and surface functionalization. Within biological systems, CDs facilitate tissue repair through multifaceted mechanisms: they restore cellular functions by enhancing migration, proliferation, and differentiation while suppressing senescence and apoptosis; simultaneously, they modulate the injured microenvironment *via* antioxidant and immunoregulatory effects. Despite growing research, a comprehensive review detailing the roles of CDs in tissue repair remains unavailable. This review addresses this gap by systematically summarizing CDs’ applications across various tissue types and elucidating their underlying mechanisms. Additionally, it critiques current research limitations and provides future perspectives, offering both theoretical insights and practical guidance for advancing CDs-based repair materials.

## 1 Introduction

Carbon dots (CDs) are zero-dimensional carbon-based nanomaterials under 10 nm in size, primarily composed of a core of sp^2^/sp^3^ hybridized carbon atoms surrounded by a surface rich in functional groups such as hydroxyl, carboxyl, and amino moieties (Xia C. et al., 2019). They can be produced *via top-down* approaches that cleave larger carbon materials like graphite, carbon nanotubes, and activated carbon, yielding structures such as graphene quantum dots (GQDs) and carbon nanodots (Ethordevic et al., 2022) Alternatively, *bottom-up* synthesis employs carbonization and polymerization of small organic molecules, biomass, or industrial waste through hydrothermal/solvothermal reactions, microwave irradiation, or pyrolysis, exemplified by coffee- and melatonin-derived CDs (Xu et al., 2017; Xin et al., 2024). Since their discovery in 2004, CDs have attracted major biomedical interest due to their unique physicochemical properties, including small size, chemical stability, superior optical performance, high water solubility, low toxicity, biocompatibility, and facile surface modification—attributes enabling diverse applications spanning optoelectronics, biomedicine, and chemical sensing (Liu et al., 2020). These properties are highly dependent on precursor selection, synthesis route, heteroatom doping, and surface functionalization, which together determine particle size, surface charge, colloidal stability, ROS-scavenging capacity, and cellular uptake. For example, biogenic carbon dot nanospheres have been integrated with mesoporous silica for contaminant adsorption (Parambil et al., 2024); Ag@CDs nanohybrids enable selective Fe^3+^ sensing (Ajith et al., 2022); and CDs have been reviewed as fluorescent probes for contaminant sensing and remediation (M et al., 2022). These examples illustrate how CD surface chemistry and structure can be tuned for specific applications, a principle that also guides their use in tissue repair.

Tissue repair is a vital physiological process restoring structural integrity and function following injury caused by acute or chronic trauma, inflammatory diseases, degenerative conditions, or metabolic disorders (Eming et al., 2014). The progression of repair is critically influenced by factors such as cellular dysfunction and disruptions within the microenvironment (Wang J. et al., 2024). Recently, CDs have emerged as promising agents for promoting tissue regeneration, offering advantages over other bioactive substances due to favorable bioavailability, high biocompatibility, and unique biological effects, thus demonstrating significant clinical potential in tissue repair (Liu et al., 2020). These biological effects are strongly influenced by CD physicochemical properties, including precursor chemistry, synthesis method, particle size, surface charge, heteroatom doping, and surface functionalization. For instance, nitrogen or metal doping can introduce SOD/CAT-like activity, while surface groups such as–NH_2_ or–COOH affect cellular uptake and migration. A more detailed discussion is provided in Section 3.

While several excellent reviews have summarized the optical properties, synthesis methods, sensing applications, and drug-delivery potential of CDs, most of them treat tissue repair as a minor subsection or focus on a single tissue type. To the best of our knowledge, this is the first review that systematically integrates CDs-mediated tissue repair across bone, skin, nerve, cardiovascular, muscle, cartilage, and liver tissues. More importantly, rather than simply listing applications, this review establishes a mechanistic framework linking CDs physicochemical properties—precursor selection, synthesis route, heteroatom doping, surface charge, and functionalization—to six core cellular processes (migration, proliferation, differentiation, senescence, death, and microenvironment remodeling). It further compares common and tissue-specific mechanisms, critically evaluates inconsistencies in the literature, and highlights translational barriers including biosafety, biodegradation, scale-up, and regulatory approval. We believe this integrated perspective distinguishes the present review from previous publications and provides a practical roadmap for designing next-generation CDs-based repair materials.

## 2 Applications of CDs in tissue repair

CDs have diverse applications in tissue repair, including bone, skin, nerve, cardiovascular, and other tissues, as detailed below and illustrated in Figure 1 and Table 1.

**FIGURE 1:**
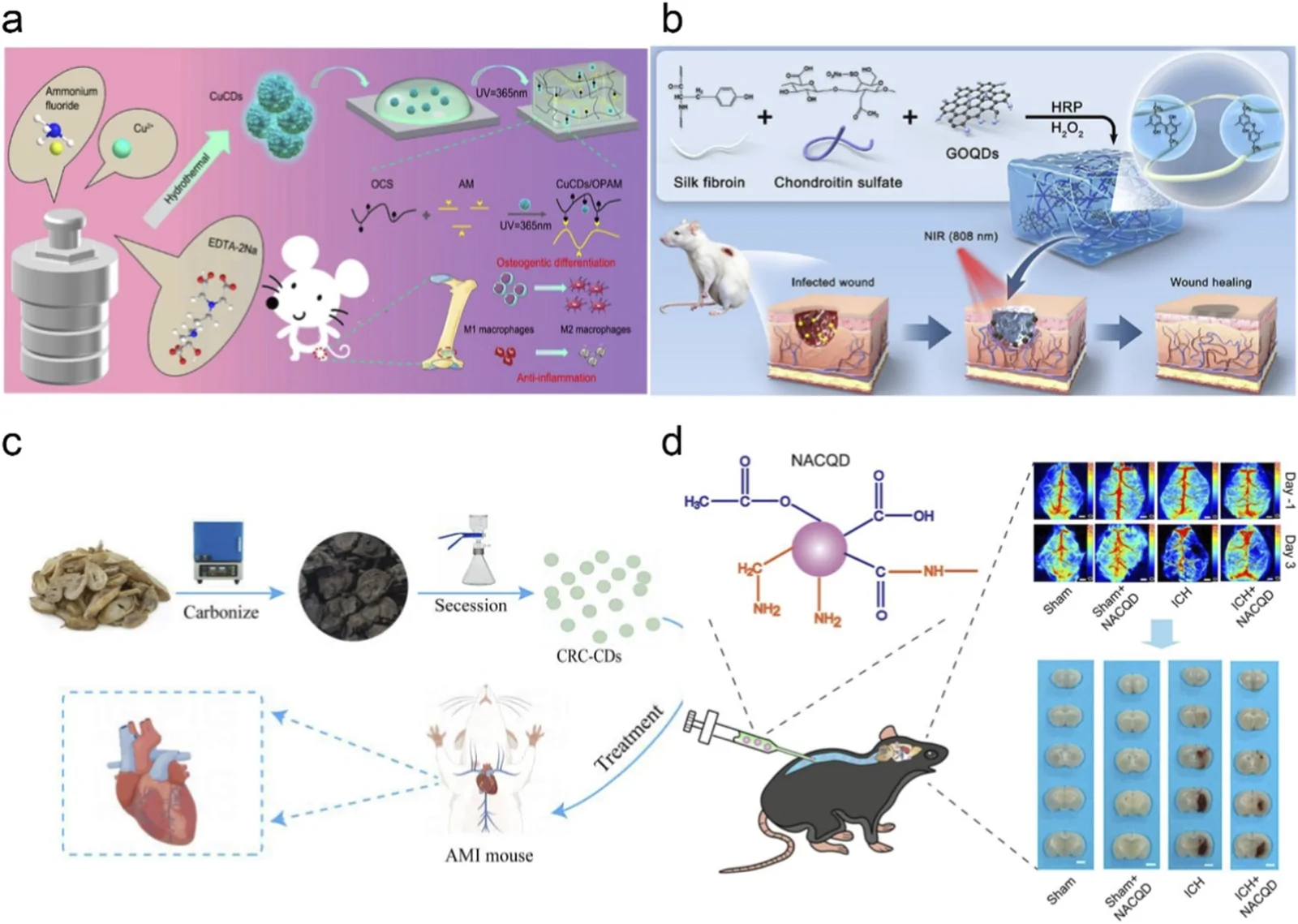
Application of CDs in **(a)** bone tissue. Reprinted with permission from Ref (Wei et al., 2024). Copyright © 2024 Royal Society of Chemistry. **(b)** skin tissue. Reprinted with permission from Ref (Deng et al., 2024a). Copyright © 2024 Elsevier. **(c)** nervous system. Reprinted with permission from Ref (Yang et al., 2023a). Copyright © 2023 American Chemical Society. **(d)** cardiovascular system. Reprinted with permission from Ref (Dong et al., 2024b). Copyright © 2022, The Author(s).

**TABLE 1:** Application of CDs in tissue repair.

CDs	Precursor	Application mode	Model	Cell types	Major finding of CDs	Limitations	Ref.
Arginine carbon dots (Arg-CDs)	L-arginine	Composite hydrogel	*in vivo* and *in vitro*	Bone mesenchymal stem cells (BMSCs) and endothelial cells (HUVECs)	Promote bone regeneration	Lack of large animal validation	(Xiao et al., 2025)
Carbon quantum dots (CQDs)	Curcumin	3D printing nanocomposite	*in vivo* and *in vitro*	Human adipose-derived stem cells (ADSCs)	Enhance bone mineralization	Lack of *in vivo* bone regeneration validation	(Nasrin et al., 2022)
Nitrogen-doped carbon dots (NCDs)	Bovine serum albumin	NCDs-HA nanoparticles	*in vivo* and *in vitro*	Osteoblasts (MC3T3-E1 cells)	Accelerate bone regeneration	Lack of mammalian validation	(Khajuria et al., 2018)
Nano Carbon Dots (CD)	Fish scales	Functionalized coating	*in vitro*	Fibroblasts (L929 cells)	Potential application in bone regeneration	Limited to *in vitro* evaluation	(Ashraf et al., 2025)
Ascorbic Acid–PEI Carbon Dots (CDs)	Ascorbic acid and polyethylenimine	Nanodelivery system and bioimaging agent	*in vivo* and *in vitro*	BMSCs, MC3T3-E1, keratinocyte (HaCaT), squamous cell carcinoma (CAL 27)	Stimulate new bone formation and repair	Lack of long-term *in vivo* toxicity data	(Bu et al., 2020)
Reduced nanographene oxide (r-nGO) carbon nanodots	Cellulose	Film	*in vitro*	Osteoblast-like cells (MG63 cells)	Bone tissue engineering application	Limited to *in vitro* evaluation	(Erdal and Hakkarainen, 2018)
Carbon dots (C-dots)	Carbon fiber	Lotion	*in vivo* and *in vitro*	HaCaT cells, fibroblasts, and HUVECs	Diabetic skin wound repair	Insufficient characteristic diabetic wound validation	(Yan et al., 2025)
Carbon nanodots (ND)	Onion peels	Composite hydrogel	*in vivo* and *in vitro*	Human amniotic membrane derived stem cells (hAMSCs)	Chronic wounds management	Lack of *in vivo* stem cell fate tracking	(Bankoti et al., 2020)
Carbon nanodots (CNDs)	Date molasses	Electrospun nanofibers	*in vivo* and *in vitro*	Primary fibroblast and keratinocyte cells	Accelerate full-thickness wound healing	Lack of healing monitoring	(Pal et al., 2017)
Carbon dots (CDs)	Citric acid and urea	Dressing	*in vivo*	-	Chemically-induced cutaneous burns therapy	Insufficient acid-burn mechanism	(Kuznietsova et al., 2025)
Folic acid-based carbon quantum dots (CQDs)	Folic acid	Delivery systems	*in vitro*	Skin fibroblasts (HFF-1)	Accelerate wound healing	Limited to *in vitro* evaluation	(Kazeminava et al., 2022)
Carbon dots (CDs)	Camelina leaves	CDs decorated Lipid droplets	*in vivo* and *in* *vitro*	Embryonic fibroblasts (NIH/3T3 cells)	Accelerate wound healing	Unverified pro-angiogenic effect	(Zhang et al., 2023a)
Fe carbon quantum dots (CQD-Fe)	Diammonium hydrogen citrate and FeCL_3_.H_2_O	Electrospun nanofibrous scaffold	*in vivo* and *in vitro*	L929 cells	Improve skin regeneration	Unclear *in vivo* iron release and clearance	(Abed et al., 2025)
Nitrogen-doped carbon quantum dots (CQDs)	Citric acid and urea	CQD-RAW nanosystem	*in vivo* and *in vitro*	Neuroblastoma cells (SH-SY5Y cells)	Alzheimer’s disease treatment	Unverified BBB recovery after NIR-induced opening	(Ye et al., 2023)
Polydopamine carbon dots (PDA C-dots)	Dopamine hydrochloride and 1,2-bis (2-aminoethoxy) ethane	Encapsulated exosomes	*in vivo* and *in vitro*	SH-SY5Y cells and brain-derived endothelial line (bEnd.3 cells)	Alleviate motor and non-motor symptoms of Parkinson’s disease	Chronic α-syn pathology unverified	(Yang et al., 2025)
Mung bean-derived carbon dots (MB-CDs)	Mung beans	Solution	*in vivo* and *in vitro*	Schwann cells	Promote peripheral nerve repair	Severe nerve defects unverified	(Zheng et al., 2025)
Aspirin-based carbon quantum dots (NACQD)	Aspirin and ethylenediamine	Solution	*in vivo* and *in vitro*	Hippocampal neuronal cells (HT22 cells)	Neuroprotection in Intracerebral Hemorrhage Mice	Invasive intrathecal delivery limits clinical translation	(Yang et al., 2023a)
Selenium-doped carbon dots (SeCDs)	L-selenocystine	Composite hydrogel	*in vivo* and *in vitro*	Neural stem cells (NSCs)	Spinal cord injury treatment	Lack of long-term fate/tumorigenicity assessment	(Qi et al., 2024)
Carbon Dots (CDs)	Glutathione	Macrophage membrane coated nanoplatform	*in vivo* and *in vitro*	Macrophages (RAW264.7) and HUVECs	Atherosclerosis therapy	Lack of thrombosis endpoint validation	(Chen et al., 2025b)
Carbon quantum dots (CQDs)	Citric acid and dimethylamine	Electroactive scaffold	*in vitro*	Myoblasts (C2C12)	Promote cardiac muscle regeneration	Lack of cardiomyocyte functional validation	(Rastegar et al., 2021)
p-phenylenediamine functionalized carbon quantum dots (modif-CQDs)	Graphite rods	Conductive cardiac patche	*in vitro*	Cardiomyoblasts (H9c2)	Cardiac tissue engineering and nursing care application	Lack of electrical stimulation	(Yan et al., 2020)
Carbon quantum dots (CQDs)	Ammonium hydrogen citrate	Drug-loaded microspheres	*in vitro*	HUVECs	Proteins-delivery in therapeutic and tissue engineering application	Lack of *in vivo* tissue regeneration validation	(Omidi et al., 2019)
Graphene quantum dots (GQDs)	-	PEGylated solution	*in vivo*	-	Benefit for myocardial infarction-induced arrhythmias	Lack of direct mechanistic validation for anti-arrhythmic effects	(Rostamzadeh et al., 2022)
Rapa functionalized carbon dots (Rapa-CDs)	Chitosan, rapamycin and acrylamide	Solution	*in vitro*	HUVECs	Inhibit vascular cell senescence	Limited to *in vitro* evaluation	(Dong et al., 2024a)
Nitrogen-doped carbon dots (NCDs)	Monohydrate citric acid and glycine	Heterojunction film	*in vivo* and *in* *vitro*	-	Repair injured vessels and relieve inflammation reactions by noninvasive phototherapy	Lack of long-term *in* *vivo* safety validation	(Li et al., 2020a)
Curcumae Radix Carbonisata-derived Carbon Dots (CRC-CDs)	Curcumae radix carbonisata	Solution	*in vivo* and *in vitro*	H9c2 cells	Cardioprotective effect	Lack of post-AMI therapeutic validation	(Dong et al., 2024b)
Carbon quantum dots (CDs)	Citric acid	Quantum dot−drug conjugate	*in vivo* and *in vitro*	Human kidney-2 cells (HK-2)	Attenuating chemotherapeutic drug-induced acute kidney injury	Unclear targeting specificity	(Zhu et al., 2023)
Metformin-derived Carbon Dots (MCDs)	Metformin and citric acid	Solution	*in vivo* and *in vitro*	RAW264.7 cells	Treat nonalcoholic fatty liver disease and associated inflammatory bone loss	Unclear liver-targeting mechanism	(Huang et al., 2023)
Carbon quantum dots (CQDs)	Garlic peels	Solution	*in vivo* and *in vitro*	Immortalized human feeder fibroblast (IHF) cell and human Wharton Jelly-derived mesenchymal stem cells (hWJ-MSCs))	Induce chondrogenesis	Lack of *in vivo* cartilage regeneration validation	(Majood et al., 2023)
Dopamine hybridized graphene quantum dot (DAGQD)	-	Composite hydrogel	*in vivo* and *in vitro*	BMSCs and RAW264.7 cells	Restore cartilage lubrication and repair worn cartilage in rheumatoid arthritis	Unclear long-term fate and clearance	(He et al., 2025)

### 2.1 Bone tissue

In bone tissue repair, CDs are utilized in composite hydrogel scaffolds (Xiao et al., 2025), electrospun fibrous materials (Ghorghi et al., 2020), functionalized coatings (Ashraf et al., 2025), and nanodelivery systems (Bu et al., 2020). They exhibit therapeutic potential in treating bone defects, inflammatory bone loss, and periodontal regeneration (Bu et al., 2020; Jin et al., 2022; Ren et al., 2021). CDs enhance bone repair by directly promoting bone marrow mesenchymal stem cell (BMSC) proliferation and mineralization, increasing osteoblast migration, stimulating angiogenesis, and modulating the immune microenvironment. For instance, ascorbic acid-derived CDs stimulate osteogenic differentiation of BMSCs, significantly enhancing new bone formation in a mouse calvarial defect model (Bu et al., 2020). Incorporating arginine CDs into composite hydrogels facilitates coupled angiogenesis and osteogenesis by promoting neovascularization in a Sprague–Dawley rat calvarial defect model, accelerating bone healing (Xiao et al., 2025). In a mouse model of periodontitis-related inflammatory bone loss, metformin-derived pharmacologically active CDs effectively mitigate bone loss by suppressing inflammatory mediators and improving the local microenvironment (Huang et al., 2023).

### 2.2 Skin tissue

In skin tissue repair, CDs demonstrate multidimensional therapeutic potential through incorporation into functional platforms such as hydrogels (Bankoti et al., 2020), electrospun fibers (Pal et al., 2017), antibacterial dressings (Kuznietsova et al., 2025), and drug delivery systems (Kazeminava et al., 2022). Their applications target complex pathological environments including diabetic ulcers (Sun et al., 2022), burn wounds (Tang et al., 2023), chronic nonhealing wounds, and infected skin injuries (Chen S. et al., 2025). CDs promote skin repair by enhancing fibroblast and keratinocyte activity, stimulating collagen deposition, and facilitating re-epithelialization, while addressing pathological barriers such as inflammation, ischemia, and metabolic dysfunction. For example, iron-modified carbon quantum dot electrospun fiber scaffolds enhance skin regeneration in acute trauma models by increasing fibroblast proliferation and collagen synthesis (Abed et al., 2025). Composite hydrogels with sustained release of zinc single-atom-modified CDs accelerate healing of chronic diabetic wounds by promoting vascular reconstruction and suppressing oxidative stress and inflammation (Dai et al., 2024).

### 2.3 Nervous system

CDs, applied directly or incorporated into hydrogel scaffolds, show significant potential in nervous system repair, addressing complex conditions such as peripheral nerve defect reconstruction (Hoveizi, 2023), spinal cord injury repair (Qi et al., 2024), and neurological recovery post-cerebral hemorrhage (Jia et al., 2025). CDs exert cytoprotective effects by inhibiting neuronal death and promote stem cell differentiation toward neural lineages, facilitating neural network reconstruction. Concurrently, they modulate the injured microenvironment *via* antioxidant and anti-inflammatory actions, enhancing functional recovery. For example, fucoidan-derived CDs demonstrated neurorepair effects in a Parkinson’s disease model by suppressing neuronal apoptosis (Han et al., 2025). Selenium-doped CDs in a rat spinal cord injury model promoted neural stem cell differentiation into neurons and enhanced synaptogenesis. Amine-functionalized aspirin CDs (NACQDs) improved the microenvironment and neurological outcomes in mice with cerebral hemorrhage through combined iron-chelating and antioxidant mechanisms (Yang X. et al., 2023).

### 2.4 Cardiovascular system

In cardiovascular tissue engineering, CDs exhibit versatile structural properties and are formulated as degradable electroactive scaffolds (Rastegar et al., 2021), conductive cardiac patches (Yan et al., 2020), and drug-loaded microspheres (Omidi et al., 2019) to meet the dynamic demands of myocardial and vascular repair. Applications include restoring myocardial function after infarction (Rostamzadeh et al., 2022), regenerating ischemic vasculature (Xu et al., 2025), and repairing arterial endothelial injury (Khan et al., 2021). CDs enhance cardiomyocyte performance, promote angiogenesis by preventing endothelial cell senescence and restoring vascular integrity, and foster a regenerative microenvironment by inducing macrophage polarization toward reparative phenotypes and scavenging reactive oxygen species (ROS). For instance, polyethylene glycolylated GQDs improved myocardial electrical conduction, corrected ECG abnormalities, restored arterial pressure and cardiac contractility, and reduced left ventricular diastolic dysfunction in a rat myocardial infarction model (Rostamzadeh et al., 2022). Rapamycin-derived CDs directly suppressed endothelial senescence (Dong J. et al., 2024), while CDs from carbon fibers promoted angiogenesis indirectly by reducing inflammatory cytokines (TNF-α, IL-6) and enhancing M2 macrophage polarization to improve the wound milieu (Yan et al., 2025).

### 2.5 Other tissues

CDs also contribute to repairing other tissues such as muscle, cartilage, and liver. Glutathione-derived CDs significantly alleviated skeletal muscle injury and enhanced repair in a mouse model of ischemia-reperfusion injury *via* antioxidant and anti-inflammatory effects (Fan et al., 2024). Folic acid-derived CDs mitigated IL-1β-induced inflammation and oxidative stress in chondrocytes, restored proliferation, alleviated mitochondrial dysfunction, and exhibited chondroprotective effects in an osteoarthritis model induced by anterior cruciate ligament transection (Jin et al., 2022). Egg white-derived CDs ameliorated iron overload-induced nonalcoholic fatty liver disease in zebrafish by chelating iron ions to reduce ROS, inhibiting endoplasmic reticulum (ER) stress and hepatocyte apoptosis, and regulating lipid metabolism and inflammation (Yu et al., 2021).

### 2.6 Comparative analysis across tissue types

Although CDs promote repair in multiple tissues, the dominant mechanisms and optimal material parameters differ by tissue context.

In bone tissue, CDs primarily promote osteogenic differentiation of BMSCs, angiogenesis, and osteoimmunomodulation. The ideal CDs for bone repair are often doped with Ca, P, Mg, or Si, or functionalized with osteoinductive molecules, because bone regeneration requires both mineralized matrix deposition and vascularization (Wu et al., 2022a; Khajuria et al., 2018). In skin tissue, the priority is rapid re-epithelialization, collagen deposition, angiogenesis, and antibacterial activity. Therefore, CDs with strong ROS-scavenging, photothermal antibacterial, or pro-fibroblast/pro-keratinocyte effects are favored (Wang H. et al., 2024; Gujju et al., 2023). In nervous tissue, the key challenges are crossing the blood-brain barrier, inhibiting neuronal apoptosis, promoting axon growth, and modulating neuroinflammation. Consequently, ultrasmall, highly biocompatible, and neuroprotective CDs are preferred, and selenium- or fucoidan-derived CDs have shown particular promise (Han et al., 2025; Zhang et al., 2024). In cardiovascular tissue, CDs must support electrical conduction, cardiomyocyte survival, and endothelial repair while avoiding thrombogenicity. Conductive or electroactive CD composites and mitochondria-targeted CDs are therefore especially relevant (Si et al., 2020; Wang H. et al., 2026). In cartilage, CDs mainly act by protecting chondrocytes from IL-1β-induced inflammation and oxidative stress, and by restoring mitochondrial function, because cartilage has limited intrinsic repair capacity (Jin et al., 2022; Wang S. et al., 2026). In liver and other metabolic tissues, CDs often function as iron chelators, ROS scavengers, or ER stress modulators, reflecting the tissue-specific pathology of iron overload or metabolic injury (Yu et al., 2021; Cao et al., 2025).

Taken together, CDs are not a uniform class of materials with a shared set of functions; rather, their repair-related activities vary substantially with precursor, size, surface chemistry, doping, and composite formulation. Across different tissues, this material diversity intersects with distinct repair requirements, leading to different preferred mechanisms and optimal parameters.

## 3 Mechanisms by which CDs promote tissue repair

### 3.1 Repairing tissue cell function

Tissue repair relies heavily on the function of repair cells, which can be categorized into two primary groups: tissue-resident cells, such as epithelial cells and fibroblasts, and regeneration-competent cells, including MSCs and tissue-specific progenitor cells. These cells are recruited and migrate directionally to the injury site during repair. Tissue-resident cells replenish damaged populations through proliferation and secrete extracellular matrix (ECM) to fill the defect, while regeneration-competent cells facilitate functional regeneration by differentiating into specialized cell types (Pena and Martin, 2024). The efficiency of cell migration, proliferation, and differentiation directly impacts the quality of tissue repair, whereas cell senescence or excessive cell death due to injury can hinder the restoration of tissue function.

#### 3.1.1 Promotion of cell migration

Cell migration is a critical first step in tissue repair, as repair cells are directed to the injury site by chemotactic signals, forming the basis for subsequent functional recovery (Li et al., 2013). CDs influence cell migration by leveraging their unique physicochemical properties, modulating chemoattractant gradients, remodeling the ECM, and activating signaling pathways related to cell migration (Figure 2).

**FIGURE 2:**
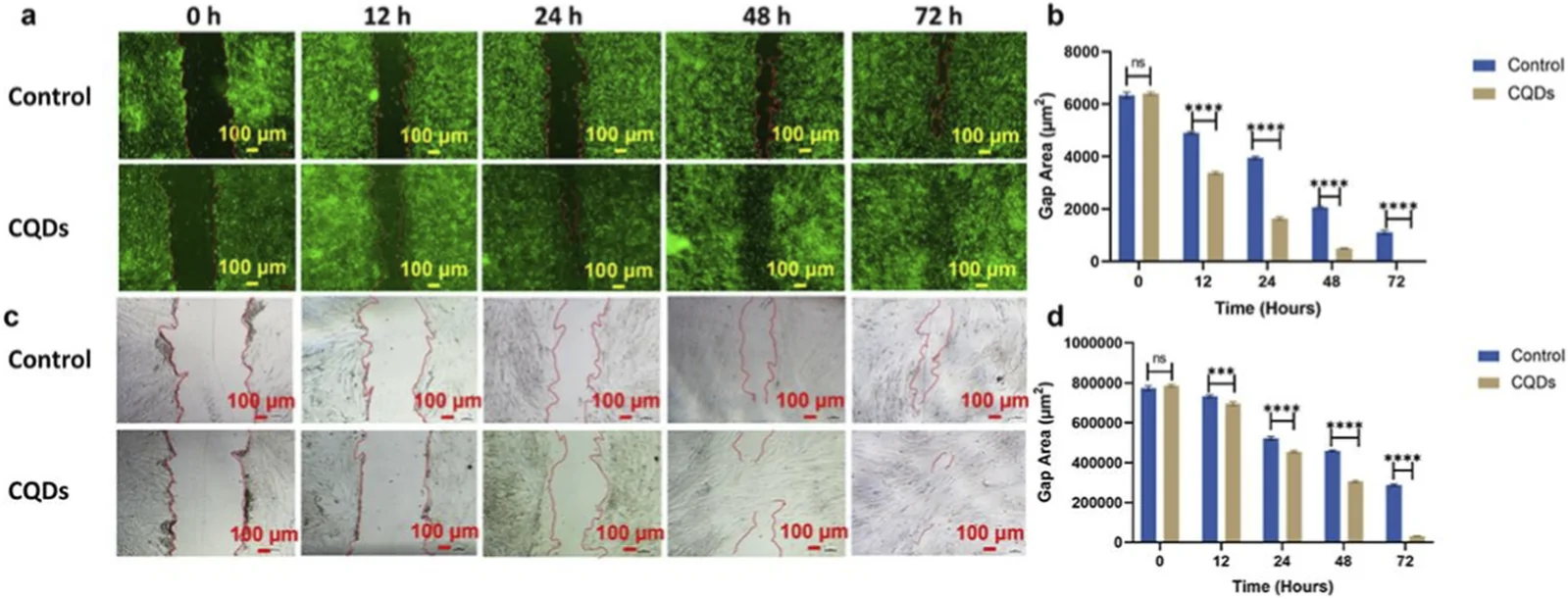
**(a–d)** Imaging and analysis of wound closure in an *in vitro* model after CQD treatment, using hWJ-MSCs. Reprinted with permission from Ref (Majood et al., 2023). Copyright © 2023 American Chemical Society.

During the hydrothermal synthesis of CDs, functional groups from their precursors can be retained on the surface through chemical bond recombination or residual bonding. These hydrophilic groups not only enhance the water solubility of the dots but also facilitate directional cell migration by forming chemical bonds and electrostatic interactions with the cell membrane (Ozyurt et al., 2023). For instance, hydrophilic groups such as hydroxyl (-OH), amino (-NH_2_), and carboxyl (-COOH) groups on the surface of bovine serum albumin CDs (BCDs) interact with the cell membrane through hydrogen bonds or van der Waals forces, promoting the migration of human skin fibroblasts (Ghataty et al., 2023). Specifically, the precise tuning of these surface chemistries—such as adjusting the ratio of carboxyl to amino groups—dictates the zeta potential and surface charge density, which directly controls the strength of cell-nanoparticle electrostatic adhesion and subsequent integrin-mediated cytoskeletal reorganization. Additionally, CDs exhibit a photothermal effect, generating localized heat under near-infrared (NIR) light irradiation. This mild thermal increase, such as raising the local temperature of the CDs in PAI/CMC/CQD multifunctional hydrogel composites to approximately 45 °C, can activate heat shock proteins, regulate cytoskeletal reorganization, upregulate cell movement-related proteins, and enhance the migration of fibroblasts and endothelial cells (Yang Y. et al., 2023). Furthermore, due to their high specific surface area and superior hydrophilicity, the incorporation of CDs into composite materials significantly promotes cell migration. Studies have demonstrated that the introduction of CDs increases the porosity of hydrogels, creating space for cell migration and nutrient diffusion. Additionally, CDs can modulate the elastic modulus and stiffness of composite materials to mimic the mechanical properties of natural bone tissue, thus promoting the migration of BMSCs through mechanosignaling pathways (Dutta et al., 2023).

Chemoattractants, including chemokines, growth factors, metabolites, and lipid mediators, play a pivotal role in regulating cell migration. Cells detect the concentration gradient of these regulatory factors through surface receptors, activating downstream signaling pathways that drive cytoskeletal reorganization and initiate migration (Ridiandries et al., 2018). Studies have demonstrated that CDs can modulate chemokine production. For example, CDs upregulate the TNF and CCL signaling pathways in CD8^+^ T cells, promoting the secretion of effector T-cell chemokines such as TNF-α and CCL5, which enhances the migration of CD8^+^ T cells, dendritic cells (DCs), and NK cells (Zhuang et al., 2025). Additionally, CDs can facilitate the migration of endothelial cells, fibroblasts, and epithelial cells by increasing the expression of growth factors like VEGFA, bFGF, and PDGF-b (Amini et al., 2025). Similarly, CDs stimulate the production of metabolites, such as ATP (Cong et al., 2023), and lipid mediators, such as PGE2 (Zhai et al., 2023), which chemotactically promote cell migration.

ECM remodeling is a critical step in tissue repair and regeneration, directly influencing cell migration. During migration, cells must traverse the ECM, and proteolytic enzymes that hydrolyze the ECM can alter its organization, making it more conducive to cell movement. This process also releases signaling molecules, such as growth factors, stored in the ECM, further regulating cell migration (Yamada and Sixt, 2019). CDs have been shown to remodel the ECM by regulating enzymes like MMP9, promoting cell migration and accelerating wound closure (Zmejkoski et al., 2022). Moreover, ECM composition changes during remodeling also impact migration. Molecules like fibronectin and laminin guide cell movement by binding to integrin receptors (Chastney et al., 2025). Studies suggest that the amino and hydroxyl groups on the surface of CDs can increase the concentration of these ECM molecules through adsorption, thereby driving directional cell migration (Pal et al., 2017).

CDs can directly influence cell migration by regulating signaling pathways related to cytoskeleton reorganization, phenotypic transformation, integrin receptors, and growth factor receptors. Dynamic cytoskeleton reorganization is a central driver of cell migration, affecting cell morphology, movement direction, and migration efficiency by controlling the spatiotemporal distribution and mechanical properties of actin microfilaments, microtubules, and intermediate filaments (Conboy et al., 2024). CDs enhance the dense arrangement of F-actin, regulate cell polarity formation, and promote pseudopod extension, thereby facilitating the migration of human dermal fibroblasts (hDFs) (Moniruzzaman et al., 2022). Additionally, CDs influence cell migration by modulating phenotypic transformation. Epithelial-mesenchymal transition (EMT) is a critical process through which epithelial cells gain migratory abilities *via* gene expression changes and phenotypic remodeling (Lamouille et al., 2014). Upon treatment with CDs, epidermal cells undergo EMT, driven by phosphorylated Smad1/5, transitioning from a static epithelial state to a migratory mesenchymal phenotype (Du et al., 2025). Integrins play a pivotal role in linking the cytoskeleton to the ECM, generating the mechanical tension necessary for migration (Pang et al., 2023). Activation of integrin signaling is another key mechanism by which CDs promote cell migration. In vascular endothelial cells, CDs stimulate migration by upregulating integrin receptor pathway genes, including ITGAV and FN1 (Tang et al., 2023). Moreover, CDs activate growth factor-mediated pathways, such as the MAPK signaling pathway and the PI3K/Akt pathway. These pathways serve as downstream effector modules, integrating cytoskeleton reorganization, matrix degradation, and chemotactic signals to drive cell migration (Liu et al., 2024).

In summary, CDs can enhance cell migration through various mechanisms, thereby promoting tissue repair. Several aspects warrant further investigation. During local tissue repair, different cell types exhibit distinct responses to migration signals. For instance, fibroblasts exhibit a marked increase in migration speed and extension area when responding to these signals, while MSCs typically form aggregates and migrate directionally along gradients. Endothelial cells, on the other hand, show a relatively mild acceleration in movement but are more likely to induce matrix remodeling and form lumen-like structures (Sahai et al., 2020; Fu et al., 2019; Liu et al., 2021). Investigating the differential effects of CDs on the migration behavior of various cell types could help optimize their performance and expand their applicability across different tissues. Furthermore, based on the distinct response modes of various cell types and the varying migration speeds required for tissue repair, CDs with specific diameters, surface charges, and degrees of functionalization could be selected. Tailored application methods, including varying concentrations, release rates, and degradation gradients, can be developed to optimize the migration speed of different cell types. This would allow for sequential guidance, enabling cells to migrate in a specific order, such as stimulating endothelial cells to migrate before fibroblasts, simulating the natural wound healing sequence and accelerating tissue regeneration.

#### 3.1.2 Promotion of cell proliferation

Cell proliferation is the central force driving tissue repair. Proliferation of resident cells accelerates the healing of damaged tissues, while stem cell proliferation sustains the regenerative cell pool, supporting subsequent differentiation and matrix deposition (Pena and Martin, 2024). The initiation of the cell cycle is a prerequisite for promoting cell proliferation, and its activation heavily relies on pro-proliferative genes such as Cyclin D1 and c-Myc. Cyclin D1 is essential for facilitating the transition from the G1 phase to the S phase and initiating S-phase gene transcription, while c-Myc directly activates pro-proliferative genes like Cyclin D1 and CDK4 and inhibits CDK inhibitors, producing a dual pro-proliferative effect (Dong et al., 2014).

CDs regulate pro-proliferative genes through multiple molecular mechanisms to drive cell proliferation:

1. Growth factor-related signaling pathways mediated by MAPK and PI3K/AKT. Both pathways are activated upon stimulation by growth factors such as VEGF. The MAPK pathway phosphorylates transcription factors like c-Myc *via* ERK, inducing cyclin expression, including Cyclin D1, while the PI3K pathway inhibits GSK3β through AKT phosphorylation, reducing Cyclin D1 degradation. During tissue repair, CDs enhance growth factor expression (Cheng et al., 2023; Cao et al., 2024), thereby activating these pathways to promote proliferation. Additionally, CDs can directly activate the PI3K/AKT pathway by phosphorylating PI3K and AKT, accelerating wound closure through enhanced proliferation (Zhang et al., 2025a).
2. Transcription factor-driven pathways such as Wnt/β-catenin and TGF-β/SMAD. These pathways activate downstream pro-proliferative genes, including c-Myc and Cyclin D1, facilitating G1/S phase transition and promoting DNA replication and cell division. Studies demonstrate that CDs treatment of deciduous tooth stem cells significantly upregulates β-catenin at RNA and protein levels, indicating activation of the Wnt/β-catenin pathway to stimulate proliferation (Gao et al., 2023). Furthermore, BMP signaling promotes MSC proliferation *via* SMAD1/5/8 activation; CDs increase p-Smad1/5/8 protein levels, activating BMP signaling and enhancing osteoblast proliferation (Khajuria et al., 2018).

Extracellular signal alterations induced by CDs can initiate intracellular signaling cascades that regulate the cell cycle. One mechanism involves the substantial enhancement of ECM compressive modulus and mechanical stiffness by CDs (Lu et al., 2019). Increased matrix rigidity promotes integrin clustering at the cell surface, which in turn facilitates the aggregation of intracellular focal adhesion proteins, leading to the enlargement, stabilization, and maturation of focal adhesions (Elosegui-Artola et al., 2016). This integrin–focal adhesion complex activates downstream mechanosensitive ion channels and triggers transcriptional programs *via* YAP/TAZ, resulting in the upregulation of pro-proliferative genes such as Cyclin D1 and c-Myc, thereby driving cell proliferation (Zhong et al., 2023). In parallel, the surface functional group composition of CDs determines their charge polarity. Positively charged CDs can electrostatically bind to cell membranes or intracellular proteins, altering protein secondary structure by increasing α-helix content while reducing β-sheet content, which facilitates the activation of functional proteins (Figure 3) (Nesic et al., 2024). Activated proteins, including growth factor receptors, subsequently stimulate downstream proliferation-associated pathways such as MAPK, leading to phosphorylation of transcription factors (e.g., c-Myc), induction of cyclins (e.g., Cyclin D1), G1/S phase transition, and enhanced cell proliferation (Yang et al., 2017). Furthermore, the photothermal properties of CDs provide an additional regulatory modality. Upon NIR laser irradiation, localized mild hyperthermia induces a heat shock response that elevates expression of growth factors such as VEGF and TGF-β, further promoting cell proliferation through PI3K/AKT, MAPK/ERK, and related signaling pathways (Liu et al., 2022a).

**FIGURE 3:**
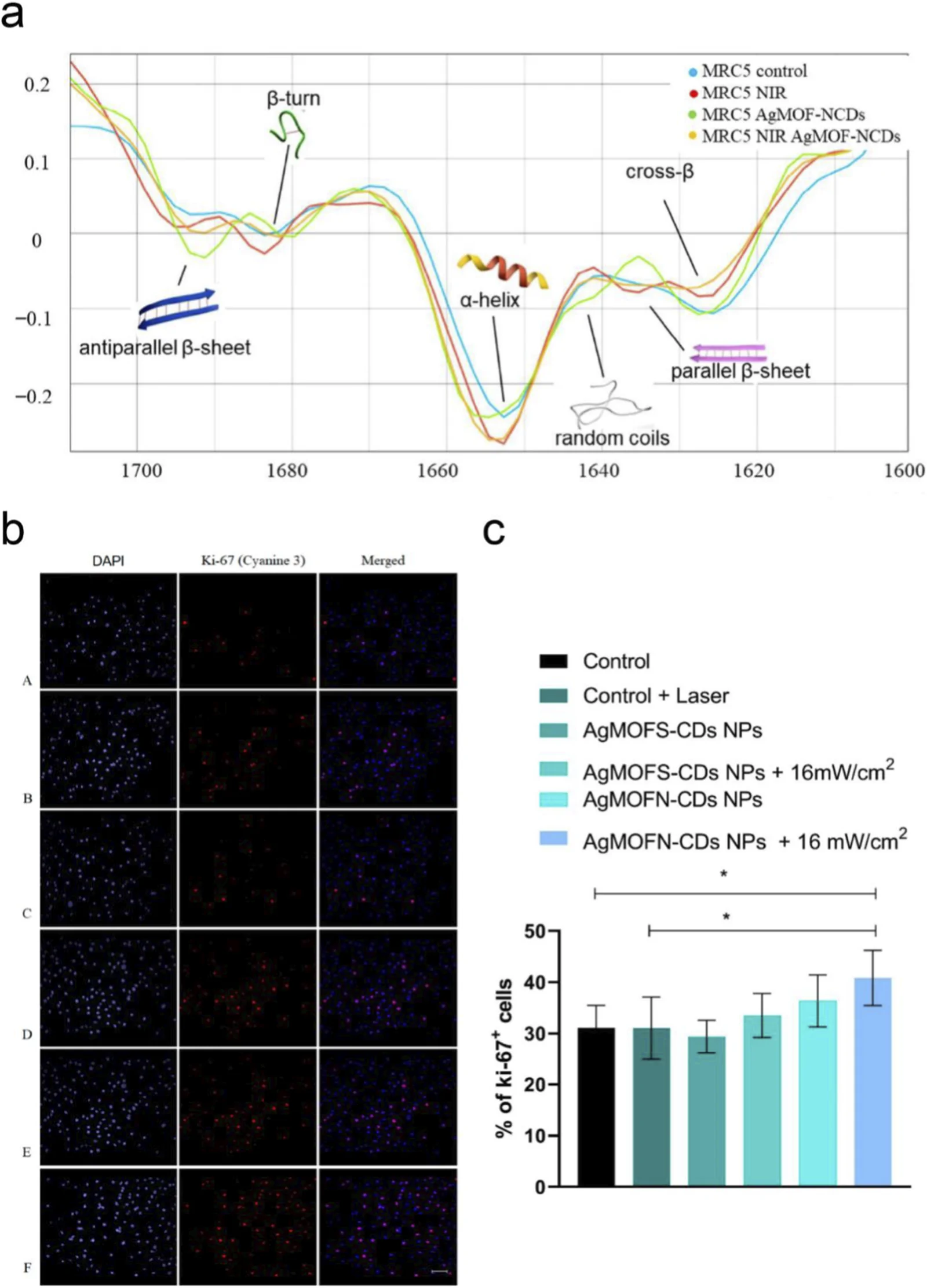
**(a)** The second derivative of the Amide I band (1500–1700 cm^−1^) of the untreated control cells (blue line) and cells treated with AgMOFN-CDs (green line), illuminated with NIR light (red line), and AgMOFN-CDs-treated and illuminated cells (combined treatment, orange line) with indicated secondary structures. **(b)** Ki-67 immunofluorescent staining of control (untreated) MRC-5 cells. **(c)** The values of proliferation index of fibroblast cells treated with AgMOFS-CDs and AgMOFN-CDs NPs without and with a laser intensity of 16 mW/cm^2^ within 3 min. Reprinted with permission from Ref (Nesic et al., 2024). Copyright © 2024, The Author(s).

CDs exhibit distinct effects on cell proliferation depending on the target cell type. Research indicates that CDs can effectively inhibit proliferation in tumor cells, demonstrating dose- and time-dependent growth inhibition across various cancer cell lines (Xia J. et al., 2019). In contrast, in tissue repair-related fibroblasts or MSCs, CDs significantly enhance proliferation, thereby accelerating wound healing and tissue regeneration (Farshidfar et al., 2023). These differing outcomes are typically due to variations in response thresholds and dose sensitivity. Tumor cells generally require higher concentrations (≥250–300 μg/mL) to achieve significant inhibition of proliferation (Wang et al., 2023), while repair cells respond favorably to lower concentrations (≤100 μg/mL) (Pal et al., 2017). A deeper understanding of how CDs influence the proliferation of different cell types could further elucidate their specific roles and optimize their applications. For instance, sustained release of low-dose, proliferation-enhancing CDs in healthy or repair tissues could be paired with targeted delivery of high-dose, cytotoxic CDs to tumor regions. Sequential administration could also be employed, first activating antitumor CDs in the tumor using carriers, followed by the infusion of repair-promoting CDs after tumor shrinkage, synchronizing with wound healing or transplant repair. Further exploration into the mechanisms and applications of CDs in cell proliferation is essential for maximizing their therapeutic potential.

#### 3.1.3 Promotion of cell differentiation

Cell differentiation plays a pivotal role in tissue repair by driving undifferentiated stem or progenitor cells to mature into the specific functional cells needed at the repair site, thereby restoring the tissue’s structure and physiological function. The precise regulation of this process directly influences the maturity and long-term stability of regenerated tissues (Lu et al., 2019). CDs modulate cell differentiation through the regulation of lineage-specific transcription factors, signal transduction pathways, autophagy, organelle function, and homeostasis.

The differentiation of stem or progenitor cells into specialized lineages, such as osteogenic, erythroid, and neural cells, is governed by lineage-specific transcription factors, which are critical for various tissue repair processes. These transcription factors, core proteins that direct differentiation, activate or repress gene expression networks by recognizing specific DNA sequences to initiate differentiation (Zaret and Carroll, 2011). Runx2, for example, is a master transcription factor for osteogenic differentiation. It directs MSCs to differentiate into osteoblasts and serves as a key regulator for bone formation and mineralization (Zhu et al., 2024). CDs have been shown to significantly elevate Runx2 levels, enhancing osteogenic differentiation and accelerating bone defect repair (Hong et al., 2025; Wu et al., 2022b). Similarly, GATA1, the master transcription factor for erythroid and megakaryocytic differentiation, plays a pivotal role in blood system regeneration. By activating hemoglobin genes, such as globin, GATA1 drives the differentiation of hematopoietic progenitor cells into mature red blood cells (Gutierrez et al., 2020). CDs derived from donkey-hide gelatin significantly promote GATA1 expression, thereby enhancing the differentiation of hematopoietic stem cells into red blood cells and aiding in the restoration of hematopoietic function in mice with aplastic anemia (Figure 4) (Wang et al., 2025a).

**FIGURE 4:**
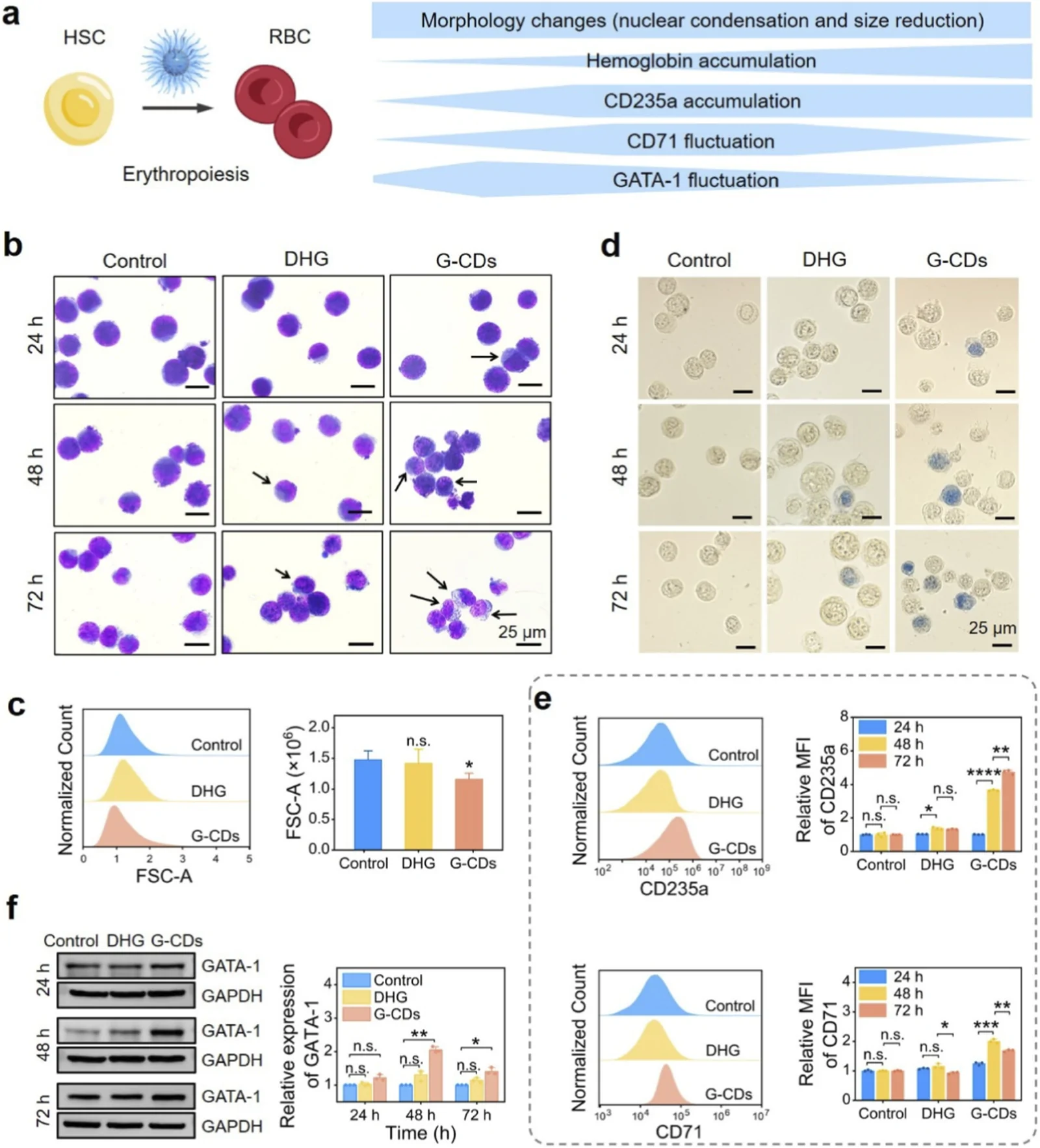
*In vitro* erythropoiesis activation performance of G-CDs. **(a)** Schematic representation of the distinctive cellular characteristics during erythropoiesis. **(b)** Wright-Giemsa staining images of K562 cells during 72 h of different treatments to indicate their morphological changes. **(c)** Average FSC of K562 cells detected by flow cytometry on the 72nd of different treatments to indicate their size distribution. **(d)** Benzidine staining images of K562 cells during 72 h of different treatments to show intracellular HGB. **(e)** The expression of CD235a and CD71 on the surface of K562 cells during 72 h of different treatments. **(f)** Western blot analysis and the quantitative histogram for GATA-1 expression during 72 h of different treatments. Reprinted with permission from Ref (Wang et al., 2025a). Copyright © 2025 American Chemical Society.

The expression of genes involved in lineage differentiation is controlled by their upstream signaling pathways. Lineage-specific pathways, such as BMP, TGF-β, and Wnt/β-catenin, regulate the expression of osteogenic-related genes like Runx2, OPN, and OCN during osteogenic differentiation (Wu et al., 2021). Nonspecific pathways, such as MAPK, can activate key factors involved in osteogenic differentiation, including Runx2, MyoD (for myogenesis), and CREB (for neurogenesis), through phosphorylation, thereby promoting the differentiation of osteoblasts, muscle cells, and neurons (Canovas and Nebreda, 2021). Research has shown that certain CDs, such as GQDs, promote osteogenic differentiation of MSCs by modulating the BMP/TGF-β pathway. Similarly, CDs derived from clove residues stimulate osteogenic differentiation and bone regeneration in mouse calvarial defects *via* the Wnt/β-catenin pathway (Hong et al., 2025; Qiu et al., 2016). Additionally, graphene oxide quantum dots have been found to enhance endothelial cell differentiation and type H blood vessel formation through the phosphorylation of ERK (Deng et al., 2024a).

Autophagy, a self-renewal process, plays a critical role in regulating the energy and raw materials necessary for the differentiation of cells. By controlling the turnover of intracellular components, autophagy can modulate the activity of differentiation-related signaling pathways, facilitate environmental transformation within the cell, and promote entry into specific differentiation states (Perrotta et al., 2020). For instance, CDs support the differentiation of human dental pulp stem cells (hDPSCs) into odontoblasts by activating autophagic flux, upregulating dentinogenesis-related genes like DMP1, DSPP, RUNX2, and SP7, and driving their differentiation (Lu et al., 2022). Moreover, CDs can aid in the degradation of damaged organelles and proteins through autophagy, providing energy and amino acids that directly support ECM synthesis. This, in turn, upregulates odontogenic differentiation genes *via* ECM-integrin-mediated signaling pathways to promote the differentiation of human dental pulp stem cells (Liu et al., 2022b). Additionally, in a dental pulp stem cell differentiation model, CDs enhance autophagy levels, thereby activating the Wnt/β-catenin pathway and significantly promoting odontoblast differentiation and mineralization (Li et al., 2020b).

The function and homeostasis of organelles are vital regulatory factors influencing cell differentiation. Mitochondria, as the energy-producing centers of cells, provide essential energy for various cellular processes. Their functional state and metabolic activity directly affect the differentiation process (Wanet et al., 2015). Mitochondria maintain a dynamic balance between fusion and fission: mitochondrial fusion creates an extended network that enhances oxidative phosphorylation efficiency, supporting energy-demanding differentiation processes (Zhong et al., 2019). In contrast, mitochondrial fission keeps the mitochondria fragmented, inhibiting premature differentiation and preserving stem cell pluripotency (Wang et al., 2017). CDs can promote osteogenic differentiation of human periodontal ligament stem cells (hPDLSCs) by regulating mitochondrial dynamics, promoting fusion and inhibiting fission (An et al., 2024). Mitochondrial dysfunction leads to the accumulation of ROS, damaging cell membranes and DNA, and impairs ATP production, disrupting the energy balance required for differentiation (Lemasters, 2014). Therefore, the timely removal of dysfunctional mitochondria and the maintenance of a healthy mitochondrial pool are critical for proper differentiation. In tissue repair, enhancing mitochondrial autophagy, removing damaged mitochondria, and ensuring energy supply can facilitate differentiation (Bhatia et al., 2019). For example, the application of graphene oxide quantum dots in a dental pulp stem cell differentiation model increases mitochondrial autophagic flux by regulating the PINK1/Parkin pathway, clearing damaged mitochondria, and maintaining mitochondrial quality, thus providing energy and metabolic support for osteogenic differentiation and promoting the repair of rat calvarial defects (Yan et al., 2024).

The homeostasis of the ER also plays a significant role in cell differentiation, activating differentiation programs through precise protein output and the regulation of calcium signals (Chen et al., 2023). The ER is responsible for the maturation and modification of many key molecules involved in differentiation. For instance, bone morphogenetic proteins (BMPs) during osteogenic differentiation rely on ER chaperones for proper folding, ensuring their ability to activate downstream Runx2 transcription factors (Hang et al., 2014). As the primary calcium store within cells, the ER directly drives differentiation transcription programs through specific calcium release. In neuronal differentiation, calcium waves mediated by IP3R channels in the ER are transmitted along dendrites, activating CaMKII kinase, which phosphorylates NeuroD transcription factors (Guo et al., 2023a). However, an excessive protein folding load or increased calcium ion release can lead to imbalances in ER homeostasis, triggering ER stress (Thastrup et al., 1990; Walter and Ron, 2011). Interestingly, mild ER stress can promote cell differentiation by activating the unfolded protein response (UPR) pathway (Turishcheva et al., 2022). For example, ascorbic acid CDs can activate IP3R receptors on the ER membrane, increase intracellular calcium levels, induce ER stress, and activate the UPR, leading to the activation of the PERK-eIF2α-ATF4 signaling pathway. This cascade upregulates ATF4, promoting the expression of bone sialoprotein (BSP) and osteocalcin (OCN), ultimately driving osteogenic differentiation and bone regeneration (Jin et al., 2020).

Current research on the application of CDs primarily focuses on osteogenesis, neurogenesis, and a limited number of hematopoietic or blood lineage cells. However, cell differentiation in other lineages, such as muscle and epithelial cells, is equally essential for tissue repair and regeneration. For example, muscle tissue repair relies on the differentiation of muscle satellite cells into myoblasts, which further differentiate, fuse to form myotubes, and mature into muscle fibers with contractile functions, thus completing the structural and functional restoration of damaged muscle tissue (Sousa-Victor et al., 2022). Despite this, there is a lack of studies on the role of CDs in the differentiation of these cell lineages. Furthermore, most existing research has primarily concentrated on changes in classic pathway molecules related to lineage differentiation mechanisms, with limited exploration into the direct targets of CDs and their regulation of downstream differentiation-related genes. Future studies should focus on identifying key targets and direct mechanisms by which CDs promote cell differentiation in diverse lineage scenarios, providing a robust theoretical foundation for their applications.

#### 3.1.4 Inhibiting cellular senescence

Tissue damage can induce cellular senescence, a state in which cells secrete senescence-associated secretory phenotype (SASP) factors, such as inflammatory cytokines and matrix metalloproteinases. These factors contribute to chronic local inflammation, tissue fibrosis, and disruption of the microenvironment, all of which hinder tissue recovery and function (Wang B. et al., 2024). CDs can target the cellular senescence process through various mechanisms, including signaling pathway modulation, regulation of SASP levels, epigenetic modifications, and clearance of misfolded proteins, thus improving tissue repair following injury.

The p53/p21 and p16 (INK4a) pathways are the primary regulators of cellular senescence. The p53/p21 pathway initiates the senescence program in response to acute stress or genomic damage, while the p16 (INK4a) pathway responds to chronic stress, maintaining cell cycle arrest and reinforcing the senescent state (Chandrasekaran et al., 2017). Overactivation of the mTOR pathway has been shown to activate both p53/p21 and p16 (INK4a) pathways, promoting and maintaining senescence (Herranz and Gil, 2018). Studies indicate that rapamycin-based CDs can target the mTOR pathway to inhibit excessive activation, reduce the expression of p53/p21 and p16 (INK4a), and mitigate cellular senescence in human umbilical vein endothelial cells (Dong J. et al., 2024).

Uncontrolled and persistent SASP plays a pivotal role in maintaining and propagating the senescent state, which impedes tissue repair (Ritschka et al., 2017). NF-κB is the key regulator of SASP, driving the production of SASP factors by directly activating transcription. CDs can intervene in the NF-κB signaling cascade, inhibit the phosphorylation and degradation of the IκBα inhibitor, reduce NF-κB nuclear translocation, and significantly lower the levels of typical SASP factors. This action alleviates chronic inflammation and counters the harmful effects of senescent cells, thereby facilitating tissue repair (Sharma et al., 2023).

Epigenetic modification of key regulatory molecules in senescence-related pathways by CDs is another key mechanism for regulating cellular senescence. Guo Z et al. reported that CDs derived from wolfberry (Lycium barbarum) significantly inhibit radiation-induced senescence in BMSCs, thereby promoting the repair of radiation-induced bone damage and recovery of bone mineral density. Their study revealed that CDs upregulate METTL3, which mediates m^6^A modification of the 3′UTR of Clip3 mRNA, leading to its degradation and downregulation of CLIP3 expression. This process inhibits p53/p21 pathway-mediated cellular senescence, reducing the expression of senescence markers in BMSCs, enhancing their proliferation and differentiation abilities, and accelerating bone repair (Figure 5) (Guo Z. et al., 2023).

**FIGURE 5:**
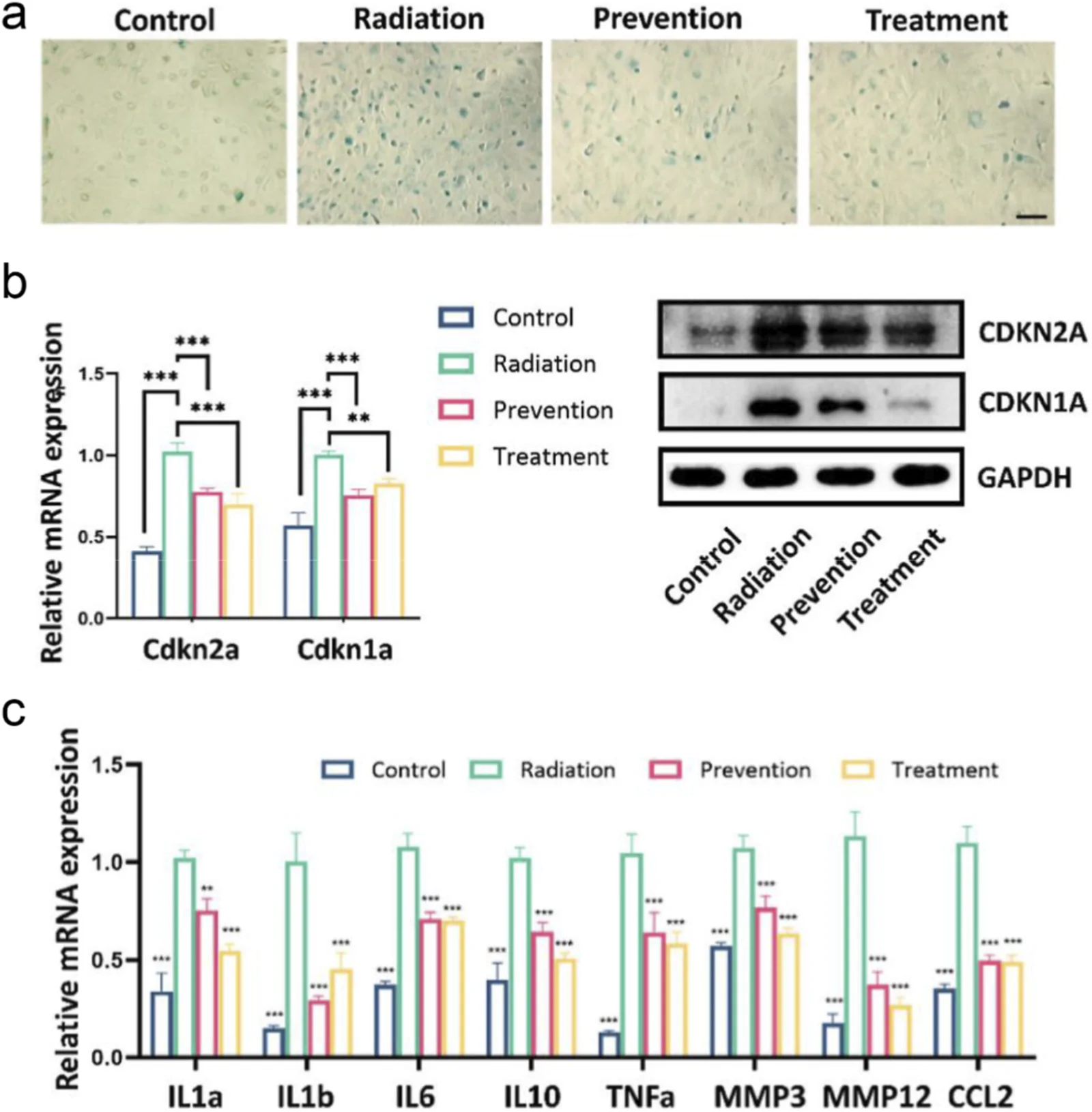
CDs attenuate radiation-induced cellular senescence of BMSCs *in vitro*. **(a)** Images of SA-*β*-gal staining indicated that CDs significantly inhibited the cellular senescence of irradiated BMSCs (*n* = 3; scale bars: 100 nm). **(b)** The expression level of CDKN1A and CDKN2A was decreased in BMSCs treated with CDs. **(c)** mRNA expression level of SASP genes inhibited by CDs in irradiated BMSCs (*n* = 3). Reprinted with permission from Ref (Guo Z. et al., 2023). Copyright © 2023 American Chemical Society.

Cellular senescence can also be induced by protein toxicity resulting from the accumulation of unfolded or misfolded proteins. For example, chronic inflammation in conditions like rheumatoid arthritis can trigger the accumulation of serum amyloid A (SAA) in extrahepatic tissues, leading to organ dysfunction and local cellular senescence (N and akamura, 2011). Following traumatic brain injury (TBI), β-amyloid (Aβ) plaques and intracellular deposits may form in brain tissue, accelerating neurodegenerative processes and inducing premature senescence of nerve cells (Scott et al., 2016). Studies have shown that CDs can directly bind to Aβ oligomers *via* charge adsorption and hydrogen bonding, reducing β-sheet formation and inhibiting Aβ self-aggregation, thereby preventing the senescence induced by Aβ accumulation (Liu et al., 2023a).

Notably, appropriate cellular senescence during pathological processes can have protective and regulatory effects. For instance, the senescence of activated fibroblasts or myofibroblasts during skin healing is a key mechanism in preventing uncontrolled fibrosis (Jun and Lau, 2022). However, the senescence of many tissue parenchymal cells or stem cells permanently impairs their ability to proliferate, repair, or differentiate, leading to exhaustion of regenerative potential and accelerating tissue dysfunction (Jeon et al., 2017). Different intervention strategies are required for the senescence of different cell types at various stages. Therefore, the optimal therapeutic goal when using CDs is to precisely inhibit or block harmful senescence while allowing beneficial senescence to play a transient role at appropriate times.

#### 3.1.5 Inhibiting cell death

Tissue damage often triggers various forms of cell death, including programmed cell death and necrosis (Park et al., 2023). Inhibiting cell death at the early stages of injury helps preserve functional cells, reduce inflammation and secondary damage, and create favorable conditions for tissue repair (Dhani et al., 2021). Consequently, CDs can significantly enhance tissue repair by targeting key death pathways.

The role of CDs in programmed cell death is outlined as follows:

1. Apoptosis: Apoptosis is a genetically regulated form of programmed cell death. Excessive apoptosis results in massive cell loss, matrix destruction, and insufficient growth factor supply, which inhibits stem cell recruitment, angiogenesis, and matrix remodeling, ultimately delaying tissue repair (Justynski et al., 2023). CDs can modulate apoptosis by downregulating the apoptotic protein Bax, inhibiting Caspase 3/9 activation, and upregulating Bcl-2. This inhibition of apoptosis promotes tissue repair. For example, Graphene quantum dots can inhibit the mitochondria-dependent apoptotic pathway in H_2_O_2_-treated SGC-7901 cells, thereby reducing cell damage (Figure 6) (Zhang R. et al., 2025).
2. Ferroptosis: Ferroptosis is a form of programmed cell death dependent on iron ions and triggered by lipid peroxide accumulation, which can lead to cell membrane rupture, amplify inflammatory responses, and hinder tissue repair and functional recovery (Stockwell, 2022). CDs can effectively inhibit ferroptosis by reducing lipid peroxidation, chelating free Fe^2+^ ions, restoring GPX4 function, and promoting GSH synthesis. For example, mung bean CDs inhibit ferroptosis in Schwann cells by blocking lipid peroxidation, significantly enhancing functional recovery after peripheral nerve injury (Zheng et al., 2025). Additionally, natural CDs synthesized from magnetite and traditional Chinese medicine “Yaoqu” (medicated leaven) inhibit iron-dependent apoptosis by chelating excess Fe^2+^/Fe^3+^ and restoring iron homeostasis, thus improving epithelial barrier damage and inflammatory responses in a DSS-induced mouse model of ulcerative colitis (Wu et al., 2024).
3. NETosis: NETosis is a specialized form of cell death in which neutrophils release extracellular traps. In conditions like rheumatoid arthritis and sepsis, overactivation of NETosis can exacerbate local inflammation and impede tissue repair (Papayannopoulos, 2018). Recent studies have shown that iron-doped CDs can downregulate the expression of key NETosis proteins, such as MPO, PAD4, and Cit H3, thereby significantly inhibiting NETosis and reducing persistent inflammatory damage. This action accelerates the remodeling of the damaged tissue matrix and microvascular formation in a rheumatoid arthritis model, creating a favorable environment for synovial repair and cartilage regeneration (Wang H. et al., 2025).
4. Pyroptosis: Pyroptosis is a form of programmed cell death mediated by inflammasome activation and gasdermin proteins. Excessive pyroptosis results in the excessive loss of epithelial, endothelial, or immune cells, destroying tissue barriers, exacerbating local inflammation, and hindering the repair process (Liu Z. et al., 2023). CDs can effectively inhibit pyroptosis and promote tissue repair. For instance, arginine-derived CDs regulate the NLRP3/Caspase 1/GSDMD-mediated pyroptosis pathway in the liver, inhibiting hepatocyte pyroptosis and alleviating liver damage in mice (Cao et al., 2025).

**FIGURE 6:**
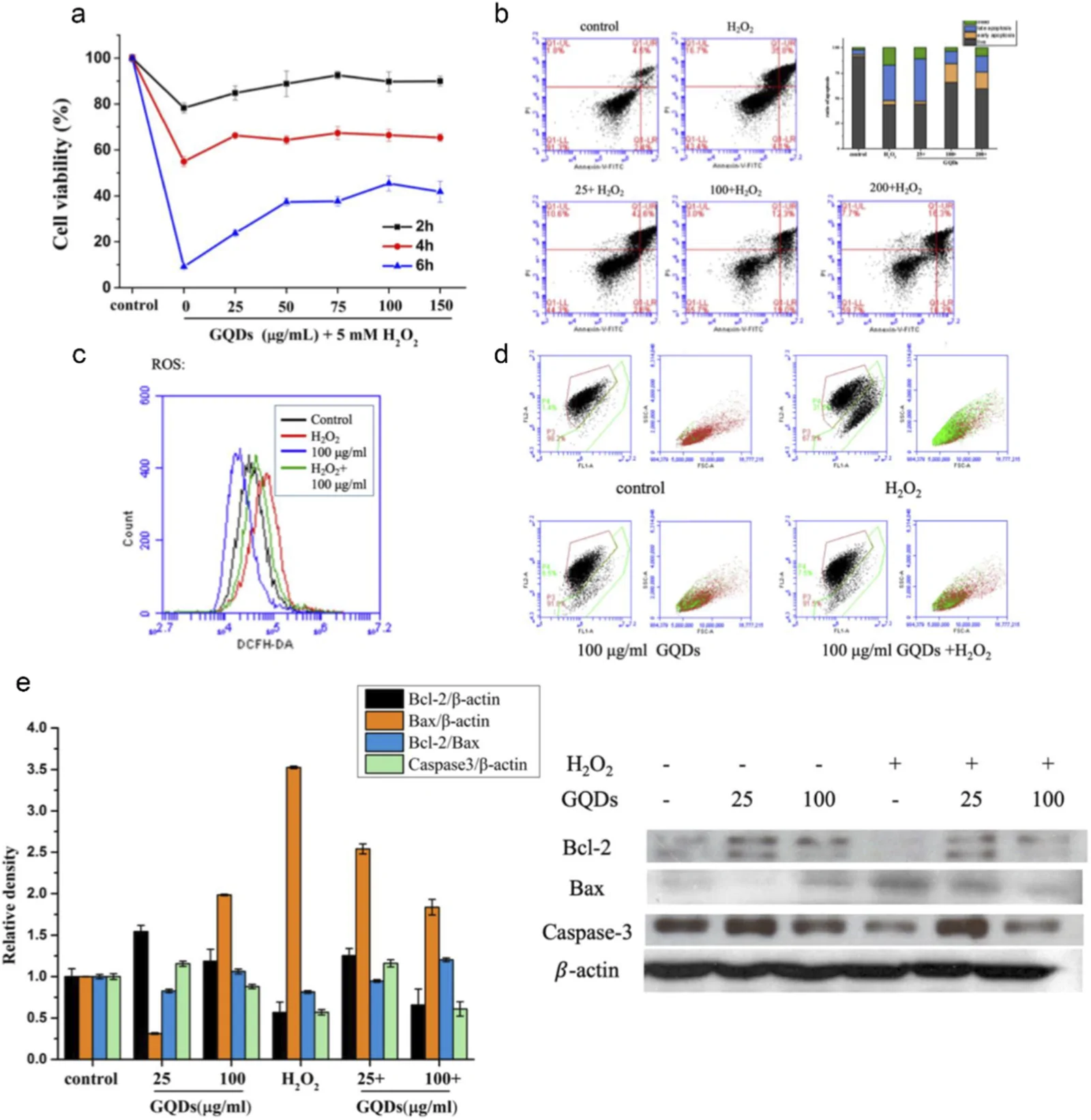
Protection against H_2_O_2_-induced apoptosis by treatment with GQDs. **(a)** Cytoprotective effects of GQDs on H_2_O_2_-induced toxicity in SGC-7901 cells. Cells were preincubated with different concentrations of GQDs for 24 h, and treated with 5 mM H_2_O_2_ for another 2, 4 and 6 h **(b–d)** The cells were incubated with GQDs for 24 h before treatment with H_2_O_2_, staining with **(b)** DCFH-DA **(c)** or Annexin V-FITC/PI **(d)** or JC-1. **(e)** Impact of GQDs on apoptosis-related protein expression in H_2_O_2_-treated cells by Western blot analysis. Reprinted with permission from Ref (Hua et al., 2019). Copyright © 2019 American Chemical Society.

Necrosis is a non-programmed form of cell death typically triggered by severe external stimuli. During necrosis, cells undergo edema, swelling of the ER and mitochondria, followed by the loss of membrane integrity, organelle disintegration, and the release of a large amount of intracellular content into the extracellular space. This process leads to excessive immune cell recruitment and degradation of the ECM (Park et al., 2023). CDs can significantly reduce cell necrosis by eliminating stimuli, inhibiting Ca^2+^ overload, and stabilizing mitochondrial function. For instance, CDs derived from Sophora flavescens roots inhibit ethanol-induced necrosis in GES-1 gastric epithelial cells by downregulating NF-κB, thereby suppressing proinflammatory factors and scavenging ROS (Hu et al., 2021). Similarly, CDs derived from Curcumae Radix significantly enhance the activity of Na^+^/K^+^-ATPase and Ca^2+^/Mg^2+^-ATPase on myocardial cell membranes, preventing isoproterenol-induced Ca^2+^ overload, inhibiting myocardial cell necrosis, and exhibiting strong cardioprotective effects (Dong L. et al., 2024). Zinc-doped ascorbic acid-derived CDs improve mitochondrial function by activating the SIRT1/PGC-1α signaling pathway. This action enhances endothelial cell viability, significantly inhibits endothelial cell necrosis under hypoxic conditions, and improves limb blood perfusion and skeletal muscle regeneration in a preclinical mouse model of critical limb ischemia (CLI) (Xu et al., 2025).

In summary, by intervening in key molecules within various death pathways, CDs can inhibit excessive or harmful cell death under pathological conditions, thereby protecting tissues and reducing damage. However, due to the limited application scope of CDs, their role in other forms of cell death, such as autophagic cell death, necroptosis, and panoptosis, remains unverified. Further investigation into the regulatory mechanisms of CDs in different types of cell death will help clarify their inhibitory effects on various factors, including cell types, stimulus intensities, and microenvironments. Additionally, crosstalk exists between different cell death pathways, where inhibiting one form of death may activate another (Kist and Vucic, 2021). Therefore, in-depth research into the regulation of CDs across different cell death forms will not only reveal the complexity of CDs in regulating these pathways but also provide a theoretical foundation for developing new strategies for synergistically targeting multiple pathways with CDs, highlighting key crosstalk nodes and dynamic balance mechanisms.

### 3.2 Repairing the microenvironment

After being recruited and localized to the injury site, repair cells encounter a microenvironment influenced by factors such as excessive ROS accumulation and an abnormal immune response, which significantly impair their repair capacity and hinder tissue recovery. By modulating the microenvironment, CDs can mitigate oxidative stress-induced damage to cellular DNA, proteins, and lipids, control inflammatory responses, enhance local oxygen and nutrient availability, and provide favorable conditions for cell migration, proliferation, and differentiation, thereby promoting tissue regeneration and repair.

#### 3.2.1 Antioxidation

Regulating oxidative stress is pivotal in tissue repair, as it creates optimal conditions by reducing oxidative damage, inhibiting inflammatory cascades, and preventing apoptosis. CDs dynamically influence the oxidative stress microenvironment through various mechanisms, including direct ROS scavenging, modulation of oxidative stress-related pathways and antioxidant enzymes, and protection of mitochondrial function (Figure 7).

**FIGURE 7:**
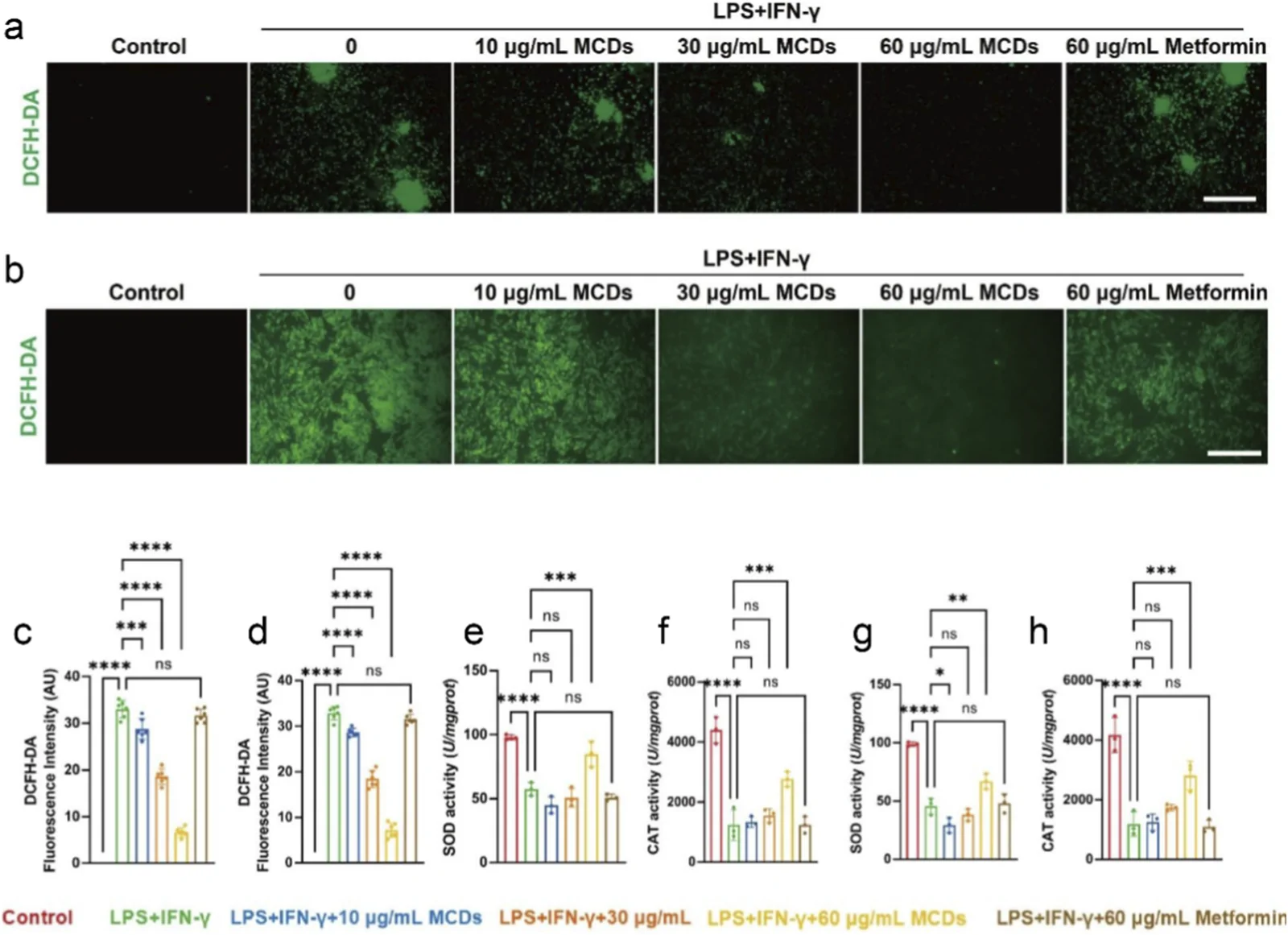
*In vitro* antioxidant ability of MCDs. **(a,b)** Representative images of DCFH-DA staining in RAW264.7 and FLS after different intervention (Scar bar = 200 μm). **(c,d)** Quantitative results of DCFH-DA fluorescence intensity in RAW264.7 and FLS from different groups. **(e,f)** The SOD and CAT activity of RAW264.7 in different groups. **(g,h)** The SOD and CAT activity of FLS in different groups. Reprinted with permission from Ref (Zhang R. et al., 2025). Copyright © 2025, The Author(s).

Following tissue injury, the levels of free radicals such as O_2_^−^·, ·OH, and H_2_O_2_ rise significantly in the local microenvironment, acting as key initiators of oxidative stress (Hunt et al., 2024). Due to their high specific surface area and abundant reactive sites, CDs possess excellent hydrogen/electron donor capabilities, thanks to functional groups like–COOH, –OH, and–NH_2_. They can donate hydrogen atoms or electrons to neutralize highly reactive free radicals such as ·OH and O_2_^−^·, transforming them into stable molecules and terminating chain reactions. Furthermore, certain functionalized CDs can effectively chelate transition metals like iron (Fe2+), thereby inhibiting the Fenton reaction and preventing the generation of highly toxic hydroxyl radicals. This multi-pathway ROS scavenging capacity holds immense clinical relevance for treating non-healing chronic diabetic wounds and ischemic tissues. This process effectively reduces the accumulation of ·OH and ·O_2_^−^ (Dong and Wang, 2023). Moreover, heteroatom doping (e.g., nitrogen, sulfur) inherently alters the electronic structure of the carbon core, lowering the bandgap and enhancing the electron-donating capacity, which is crucial for neutralizing free radicals. This doping imparts dual catalytic activities, mimicking superoxide dismutase (SOD) and catalase (CAT), enabling rapid dismutation of O_2_^−^· into H_2_O_2_, which is then further decomposed into H_2_O and O_2_, significantly enhancing H_2_O_2_ scavenging efficiency (Yan et al., 2025). Furthermore, the carbon structure of CDs, enriched with sp^2^ hybrid domains and π-conjugated systems, increases the surface electron density and promotes rapid electron migration within the nanoparticles, accelerating reactions with ROS. This further amplifies the free radical trapping capacity through π–π electron coupling and quantum confinement effects (Li et al., 2023).

The inactivation of both intracellular and extracellular antioxidant pathways, alongside decreased activity of antioxidant enzyme systems due to tissue injury, significantly impairs ROS scavenging capacity in the local microenvironment, resulting in sustained oxidative stress. Nrf2, known as the “master regulator” of cellular defense against oxidative stress, plays a pivotal role in maintaining cellular redox balance, detoxification, and metabolic adaptation (Zhang J. et al., 2025). CDs can activate the Nrf2/ARE pathway to upregulate the expression of antioxidant genes, thereby restoring the activity of endogenous antioxidant enzymes such as HO-1, NQO1, SOD, and CAT, ultimately regenerating the cellular antioxidant capacity (Cao et al., 2025; Zhou et al., 2024). Similarly, the AMPK/FoxO1 pathway upregulates the transcription of downstream antioxidant genes by activating the transcription factor FoxO1, thus maintaining cellular redox balance and enhancing overall antioxidant defense. Studies have shown that in the high oxidative stress microenvironment of acute kidney injury, selenium-doped CDs can activate AMPK to promote nuclear translocation of FoxO1, which directly drives the expression of antioxidant enzyme-encoding genes like SOD, CAT, and GPx, while inhibiting excessive ROS production (Luo et al., 2020).

Mitochondrial dysfunction plays a central role in disrupting cellular redox homeostasis and intensifying oxidative stress in the local microenvironment. Following tissue injury, mitochondrial membrane potential is lost, electron leakage in the respiratory chain increases, ATP synthesis is impaired, and mitophagy is disrupted, leading to excessive production of mitochondrial ROS (mtROS) (Zong et al., 2024). Therefore, targeting mitochondria to restore their functional homeostasis is essential for maintaining redox balance. CDs can protect mitochondrial function and reduce oxidative stress through multiple mechanisms, thereby promoting tissue repair:

1. Mitochondrial targeting and localization: Through surface modifications with triphenylphosphine (TPP) or the use of enriched guanidyl/amino groups, CDs can efficiently accumulate in the mitochondrial matrix across membranes, scavenging mtROS precisely, and restoring mitochondrial membrane potential and respiratory chain integrity (Hua et al., 2017).
2. Promotion of the balance between mitochondrial biogenesis and autophagy: Selenium-doped CDs regulate the PGC-1α/NRF1 pathway to increase mitochondrial biogenesis while moderately activating PINK1/Parkin-mediated mitophagy to clear damaged mitochondria, thereby maintaining mitochondrial homeostasis (Zhang et al., 2024).
3. Enhancing ATP production and cellular energy metabolism: The quantum confinement effects and π–π conjugated structures of CDs improve electron transport efficiency, synergistically promoting the recovery of respiratory chain complex activity. This enhances ATP synthesis, thereby providing energy support for cell proliferation and migration (Truskewycz et al., 2022).

The antioxidant function of CDs constitutes a dynamic protective system that integrates scavenging, transformation, and regulation. Enhancing the antioxidant capacity of CDs based on existing research can create more favorable conditions for tissue repair under pathological conditions such as ischemia, inflammation, and neurodegeneration. This antioxidant function can be further improved through the following strategies:

1. Regulating the selection of carbon sources/precursors: Selecting precursors containing phenolic hydroxyl, amino, sulfhydryl, or conjugated heterocycles to enhance electron transfer capacity (Son et al., 2021);
2. Increasing the specific surface area of CDs and introducing edge defects or vacancies to expose additional active sites (Zhao et al., 2023);
3. Directionally modifying antioxidant groups: Incorporating antioxidant molecules, such as polyphenols or thiols, through covalent or noncovalent binding (Wang et al., 2020);
4. Constructing cascade antioxidant systems: Combining multiple strategies to endow CDs with multienzyme activity or multifunctional integration (Zhang et al., 2023b).

#### 3.2.2 Immunomodulation

Immune cells play a pivotal role in regulating the immune microenvironment and maintaining homeostasis through processes such as secretion, phagocytosis, and antigen presentation (Pena and Martin, 2024). During tissue repair, immune cells with different phenotypes and functions collaborate to transition from inflammatory stress to regenerative remodeling (Loffredo et al., 2024). In various disease models, CDs can precisely modulate the functional state of immune cells, influence the immune microenvironment, and enhance tissue repair. The effects of CDs on different immune cells can be summarized as follows:

1. Neutrophils: In tissue injury, excessive neutrophil proliferation and infiltration at the lesion site lead to elevated local levels of proinflammatory factors, further spreading inflammation and hindering tissue healing and regeneration (Herr et al., 2022). CDs can effectively inhibit excessive neutrophil proliferation and chemotaxis, alleviating inflammatory infiltration. For example, in a collagen-induced rheumatoid arthritis mouse model, intravenous administration of Fe-doped CDs significantly reduced neutrophil infiltration in the joint synovium, decreased neutrophil autophagic flux, induced neutrophil apoptosis, inhibited NET formation, and ultimately alleviated joint inflammation and tissue damage (Wang H. et al., 2025).
2. Macrophages: In diseased lesions, macrophages often exhibit excessive M1 polarization and impaired M2 polarization, leading to severe inflammation, tissue destruction, and impaired repair (Yu et al., 2022). CDs can improve inflammation and promote tissue repair by modulating macrophage polarization and function. In an *in vitro* osteoarthritis model, folic acid-derived CDs reprogrammed LPS-induced macrophages by downregulating M1 markers and proinflammatory cytokines while upregulating M2 markers, thereby suppressing nitric oxide production. This macrophage repolarization, along with reciprocal modulation via chondrocyte–macrophage crosstalk, reduced inflammatory responses and extracellular matrix degradation, contributing to joint tissue protection in osteoarthritic lesions (Figure 8) (Jin et al., 2022).
3. DCs: During injury-related diseases, resident and infiltrating DCs rapidly mature, secrete proinflammatory factors, and migrate to lymph nodes to activate T cells, initiating adaptive immune responses. However, excessive DC maturation and activation exacerbate inflammatory responses, worsening tissue damage, and impairing tissue healing (Cabeza-Cabrerizo et al., 2021). Studies have shown that CDs can inhibit DC maturation and the resulting inflammatory cascades (Wang et al., 2021). Furthermore, CDs can induce a tolerogenic or inhibitory phenotype in DCs. For instance, after endocytosis, GQDs induce DCs to adopt a tolerogenic phenotype, reducing IL-12 secretion, increasing IL-10 production, and promoting regulatory T-cell generation, thereby facilitating tissue repair (Tomic et al., 2017).
4. T lymphocytes: In various injury-related diseases, T cells are often overactivated, skewing toward proinflammatory Th1/Th17 subsets, with a concurrent decrease in the number or function of regulatory T cells (Tregs). This imbalance exacerbates inflammation, worsens tissue damage, and delays repair (Le and e, 2018). CDs can restore immune balance and reduce tissue inflammation by modulating the Th1/Th17/Treg ratio, reducing excessive T-cell activation, and inhibiting proinflammatory cytokine secretion. For example, in a rat model of periodontitis, ε-polylysine CDs endocytosed by T cells inhibited proinflammatory Th1/Th17 cells and their secreted IFN-γ/IL-17, while increasing anti-inflammatory IL-10 and promoting Treg generation, resulting in reduced local inflammation and accelerated bone regeneration (Deng X. et al., 2024).

**FIGURE 8:**
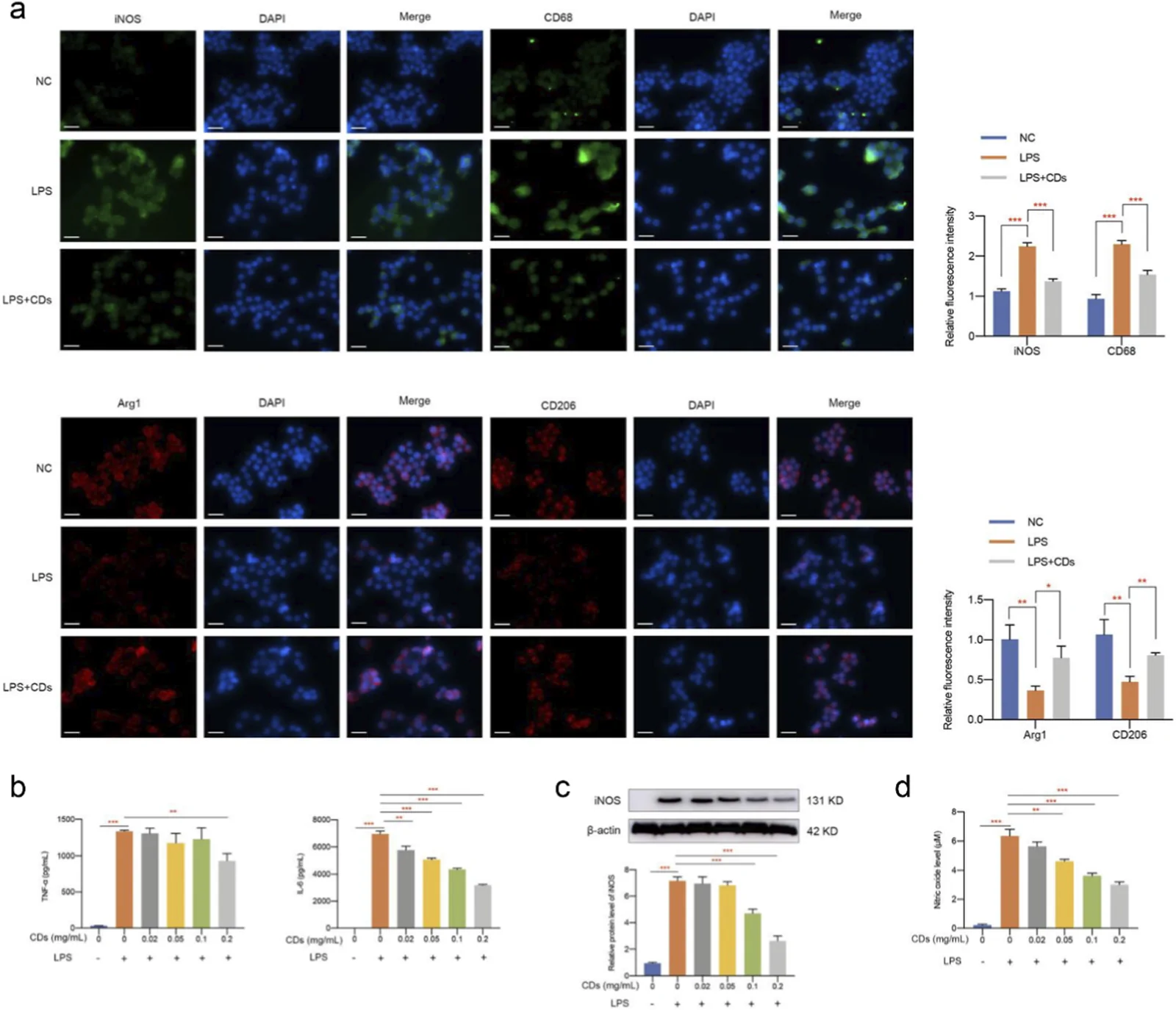
CDs orchestrated macrophage repolarization *in vitro*. **(a)** Immunofluorescence staining results indicated CDs incubation could prevent the polarization of macrophages to proinflammatory type. Scar bar: 20 μm. **(b)** The induced concentration of proinflammatory factors in LPS-induced RAW264.7 cells was suppressed by CDs treatment. **(c)** The protein level of iNOS in RAW264.7 cells under different treatment. **(d)** Griess assay was used to evaluate the NO scavenging effects of CDs. Reprinted with permission from Ref (Jin et al., 2022). Copyright © 2022, The Author(s).

In summary, CDs offer several unique advantages and characteristics in regulating immune cells. However, their regulation of immune cells often exhibits biphasic behavior: they can activate immune cells under certain conditions and inhibit them under others (Ayaz et al., 2020). This biphasicity is typically influenced by factors such as dosage, application method, CD functionalization, and the surrounding microenvironment. Reliable control over the regulatory functions of CDs can be achieved through precise drug delivery, targeted ligand-based delivery, and functionalized responsive regulation. Moving forward, the biphasic nature of CD regulation can be strategically utilized: during tissue repair, antioxidant CDs can first be employed to protect the repair microenvironment; then, small amounts of proinflammatory factors can be released at critical stages to promote macrophage polarization from M1 to M2, enhancing regenerative functions and systematically improving tissue repair and regeneration.

These immune-regulatory effects are not isolated but are embedded in a broader repair network involving oxidative stress, angiogenesis, ECM remodeling, mitochondrial function, and cell–cell communication. ROS scavenging protects mitochondrial function and energy supply, thereby supporting the migration, proliferation, and differentiation of repair cells; this is particularly relevant in myocardial infarction, where preserving cardiomyocyte and endothelial cell function is critical for electrical and contractile recovery. Immunomodulation and angiogenesis are closely coupled, and this coupling is especially prominent in bone repair, where M2 macrophage polarization promotes type H vessel formation and osteogenic differentiation. ECM remodeling is both a consequence and a regulator of these processes, as changes in matrix stiffness feed back on integrin signaling and YAP/TAZ activation; in skin wounds, this feedback helps coordinate fibroblast proliferation, collagen deposition, and re-epithelialization. Mitochondria act as a central hub linking redox balance, metabolism, and lineage commitment, and in cartilage repair, protecting chondrocyte mitochondria from IL-1β-induced dysfunction helps limit ECM degradation. Cell–cell communication through chemokines, dendritic cells, T cells, and macrophage–chondrocyte/osteoblast crosstalk further coordinates the regenerative niche. Although not every mechanism has a tissue-specific signature, these examples illustrate how the same interconnected network can produce context-dependent repair outcomes. Therefore, CDs are best viewed as network modulators, and future studies should evaluate their effects at a systems level.

## 4 Conclusions: limitations and prospects

The clinical translation and practical application of CDs currently face several challenges. These limitations can be categorized into three main areas: application direction, functional design, and mechanism research, with prospects for future development outlined below.

Most current studies on the use of CDs in tissue repair are confined to single-cell line models or single-tissue defect models, which fail to address the complexities of multitissue and multicell interactions in the dynamic *in vivo* environment. Due to the significant differences between *in vitro* models and *in vivo* microenvironments, there is a need to develop more *in vivo*-like 3D coculture systems or organoid models to explore the role of CDs at the interface of multiple tissue types. Additionally, the integration of CDs with bioprinting or microfluidic chip technologies could facilitate the construction of “multitissue-multicell” repair platforms. These platforms would enable precise delivery and functional regulation in complex repair environments, providing a foundation for the development of clinical multifunctional regenerative materials.

Current research tends to focus on a single function of CDs, such as promoting osteogenesis, angiogenesis, or antioxidation, but often overlooks the multimodal synergistic effects that are crucial for effective tissue repair. To address this, CDs can be enriched with additional functions through chemical conjugation or electrostatic adsorption, optimizing their properties and expanding their potential applications. In the future, modular multistep synthesis strategies could be employed to sequentially assemble modified molecules with distinct functions on the CDs’ surface. Additionally, the development of intelligent responsive systems, such as pH/enzyme-sensitive or light-controlled release mechanisms, could enable dynamic functional switching of CDs at different stages of tissue repair.

Although current research has focused on specific classical signaling pathways involved in the repair and treatment of particular diseases, most findings are based on phenotypic observations at the cellular level, lacking comprehensive analyses of molecular targets, downstream transcriptional networks, and cell-matrix interactions. Furthermore, there is limited *in vivo* dynamic tracking and quantitative analysis. Future research could integrate omics technologies and high-resolution imaging methods, such as *in vivo* fluorescence confocal microscopy, along with single-cell sequencing and spatial omics, to systematically elucidate the multilevel and multidimensional repair mechanisms mediated by CDs. Additionally, the use of artificial intelligence-based multiomics big data models could predict the functional profiles of CDs with varying structures and functionalizations. Key molecules could then be validated through gene editing or targeted knockout, providing a solid mechanistic foundation for the precise design and clinical application of CDs.

Before widespread clinical application can be realized, it is imperative to address existing inconsistencies in the literature, such as the biphasic dose-dependent effects where CDs may promote repair cell proliferation at low doses but induce cytotoxicity at higher concentrations. Nevertheless, clinical translation still faces several practical barriers. The long-term biosafety, biodegradation, biodistribution, pharmacokinetics, and immunogenicity of CDs remain insufficiently understood, especially after repeated or systemic administration. In addition, large-scale synthesis with consistent particle size, surface chemistry, and doping level is difficult, leading to batch-to-batch variability that complicates quality control. Finally, regulatory approval and commercialization require standardized characterization, safety evaluation, and scalable manufacturing, transitioning from small animal models to human-relevant systems, which are still lacking for CDs-based repair materials.
